# Local Anesthetic Toxicity in Epidural Analgesia for Elderly Patients: A Case Report

**DOI:** 10.1155/cria/2604746

**Published:** 2026-08-31

**Authors:** Thi Anh Tuyet Cao, Thang Toan Nguyen

**Affiliations:** ^1^ Department of Anesthesiology, Saint Paul General Hospital, Hanoi, Vietnam; ^2^ Center for Anesthesia and Surgical Intensive Care, Bach Mai Hospital, Hanoi, Vietnam, bachmai.gov.vn; ^3^ Department of Anesthesiology, Hanoi Medical University, Hanoi, Vietnam, hmu.edu.vn

**Keywords:** accumulation of local anesthetics, aged, bupivacaine 0.5%, cardiovascular collapse (CC), central nervous system (CNS), elderly patients, epidural analgesia, geriatrics, local anesthetic systemic toxicity

## Abstract

**Background:**

Elderly patients have a higher likelihood of undergoing surgical procedures compared to younger populations, a factor associated with increased perioperative mortality and anesthesia‐related morbidity. Regional anesthesia in geriatric patients offers a favorable safety profile, optimizes analgesia, and contributes to reducing postoperative complications, particularly within the framework of enhanced recovery after surgery (ERAS) protocols. Nevertheless, local anesthetic systemic toxicity (LAST) remains a rare yet potentially life‐threatening complication in the context of epidural analgesia. Its severity is amplified in scenarios involving prolonged local anesthetic infusion or in patients with elevated susceptibility due to age‐related physiological changes or comorbidities.

**Case Presentation:**

A case report details an instance of LAST induced by continuous epidural administration of bupivacaine 0.1% in a 95‐year‐old female patient following surgery for bowel obstruction. We describe the clinical presentation, diagnostic considerations, therapeutic interventions, and subsequent recovery.

**Conclusion:**

Epidural analgesia is an effective method of pain relief, providing prolonged and continuous analgesia with a relatively low risk of systemic toxicity. Nevertheless, LAST may still occur, particularly during prolonged administration or in vulnerable populations, such as elderly patients and those with hepatic, renal, or cardiovascular dysfunction. In the critical care setting, clinicians should maintain a high index of suspicion for LAST while excluding other differential diagnoses, including shock, stroke, or pulmonary embolism. Importantly, the potential accumulation of local anesthetics during epidural analgesia should be considered even when dosing adheres to current recommendations, as age‐related physiological and pharmacokinetic changes may increase susceptibility to toxicity. This case also highlights the important role of intravenous lipid emulsion (ILE) as an effective rescue therapy in the management of LAST, with rapid improvement in neurological manifestations following treatment.

## 1. Introduction

Local anesthetic systemic toxicity (LAST) is a rare but potentially life‐threatening complication of regional anesthesia. Its reported incidence is approximately 0.87%–1.8% for peripheral nerve blocks and is lower for epidural analgesia when standard dosing protocols are followed, although the true incidence is likely underestimated due to underreporting and misdiagnosis [1–3]. Bupivacaine is a long‐acting amide local anesthetic widely used for epidural and peripheral nerve block techniques. However, due to its high lipid solubility and significant cardiotoxic potential, the risk of systemic toxicity remains clinically relevant, particularly in prolonged infusions or highrisk patients [4]. The development of LAST is multifactorial, involving procedure‐related factors, such as inadvertent intravascular injection, excessive dosing, rapid absorption from highly vascular sites, and prolonged infusions, as well as pharmacokinetic factors including impaired hepatic metabolism and reduced protein binding, which increase circulating free drug concentrations. Patient‐related risk factors include advanced age, low body weight, reduced cardiac output, hepatic dysfunction, pregnancy, and hypoalbuminemia, with critically ill patients being particularly vulnerable [2]. Clinically, early recognition is essential but challenging, as initial manifestations are nonspecific and may mimic septic shock, acute myocardial infarction, pulmonary embolism, acute heart failure, or acute cerebrovascular events. Delayed diagnosis can rapidly lead to deterioration. Intravenous lipid emulsion (ILE) remains the cornerstone of treatment and has been associated with improved outcomes in severe cases.

## 2. Case Presentation

A 95‐year‐old female patient (weight: 56 kg, height: 159 cm) with a medical history of hypertension and type 2 diabetes mellitus was on regular treatment with metformin1000 mg/day and Twynsta 45 mg (telmisartan/amlodipine 40 mg/5 mg) once daily. She had undergone an appendectomy 6 years earlier. She was admitted with periumbilical abdominal pain and diagnosed with small bowel obstruction due to adhesions. Preoperative laboratory tests were mostly within normal limits, and the chest X‐ray showed no evidence of pneumonia.

Preoperative laboratory tests showed: hemoglobin 134 g/L, platelets 284 × 10^9^/L, white blood cells 10 × 10^9^/L with neutrophils 77%, creatinine 82.5 μmol/L, urea 9.2 mmol/L, aspartate aminotransferase (AST) 21 U/L, alanine aminotransferase (ALT) 10 U/L, sodium 136 mmol/L, potassium 3.98 mmol/L, and blood glucose 8.56 mmol/L (Table 1).

**TABLE 1:** Perioperative laboratory findings.

Laboratory test	Laboratory value					Reference range
Date	12/01	14/01	15/01	17/01	19/01	
HGB (g/L)	103	145	116	119	—	130–160
PLT (× 10^9^/L)	317	370	131	146	—	150–450
WBC (× 10^9^/L)	8.8	11	8.7	9.8	—	4–10
NEUT%	94	68	83	91	—	55–70
Sodium (mmol/L)	137	119	140	139	142	135–145
Potassium (mmol/L)	3.0	3.7	2.6	3.08	3.4	3.5–4.5
pH	7.43	7.42	7.48	7.48	—	7.35–7.45
HCO_3_^-^	28	18	22	28		20–24
Carbon dioxide	45	21	25	36		35–45
BE	8	−10	−4	3		(−2)–2
P/F	328	280	380	342		
Creatinine (μmol/L)	60	59	60	51.5	58	58–96
Urea (mmol/L)	8.3	1.3	5.9	7.4	9.6	1.7–8.3
AST (U/L)	59	13.7	—	218	79	< 35
ALT (U/L)	17	6.4	277	324	171	< 35
Albumin (g/L)	26.6	37.7	—	35	—	31–35
Lactate (mmol/L)	0.98	12.58	2.35	2.29	3.29	0.5–2.2
Glucose (mmol/L)		—	—	—	16	3–6.5
BNP (pg/mL)		1062	—	1913	3173	< 50
Pro‐calcitonin (ng/mL)	—	0.125	—	—	—	< 0.1
Fibrinogen (g/L)	—	91	—	5.12	—	2–4
D‐dimer (μg/mL)	—	10	—	4.8	—	< 0.5
Prothrombin (%)	92.6	91	—	84.8	—	70–140
APTT (s)	29	24	—	31.5	—	26–38

The patient underwent emergency surgery for release of an adhesive band and restoration of bowel continuity. Postoperatively, continuous epidural analgesia was administered with bupivacaine 0.1% at 4 mL/h (96 mg/day). She was extubated safely and placed on nasal oxygen at 3 L/min.

**FIGURE 1:**
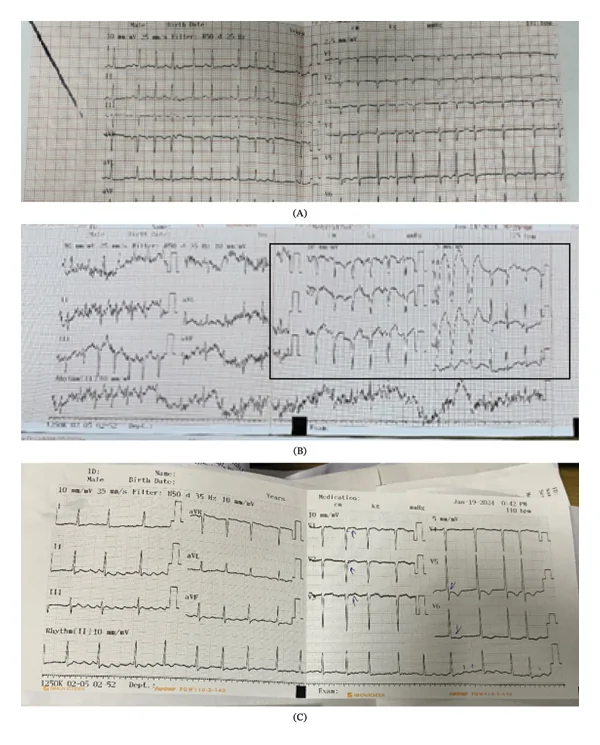
Electrocardiographic changes during the clinical course. (A) Initial electrocardiogram (ECG) performed in the emergency department showing normal sinus rhythm. (B) ECG recorded during the event demonstrating ventricular premature complexes. (C) Resolution of arrhythmia with no further ventricular ectopy.

**FIGURE 2:**
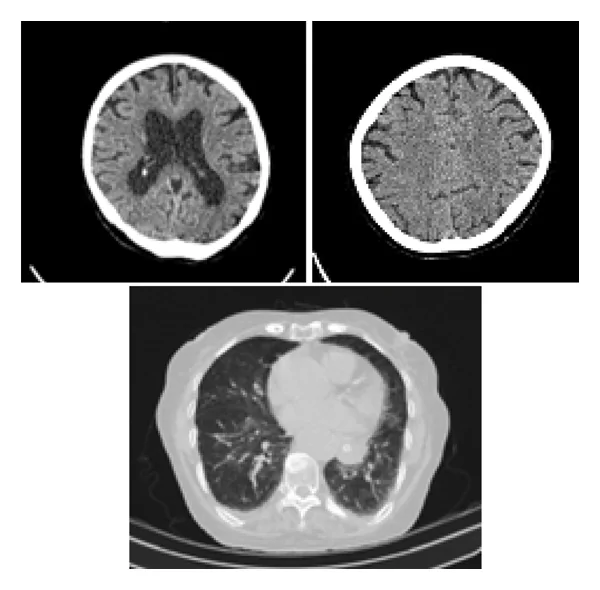
Brain CT scan showing no acute abnormalities, with no evidence of pulmonary embolism.

On postoperative Day 3, she developed acute neuropsychiatric and cardiovascular manifestations, including manic delirium, lower limb rigidity with myoclonus, inability to lift her legs, tachycardia with frequent ventricular premature contractions, and severe hypertension (180–190 mmHg). The differential diagnoses included high neuraxial block, stroke, acute myocardial infarction, and LAST. We checked the epidural catheter position to ensure that it was not located in the subarachnoid space, thereby excluding total spinal anesthesia. Fluid balance assessment showed no evidence of volume overload. On lung examination, no pathological crackles suggestive of pulmonary congestion were detected. The presence of neuromuscular hyperexcitability and arrhythmia favored the diagnosis of LAST, despite its rarity with epidural administration (Figures 1 and 2).

**FIGURE 3:**
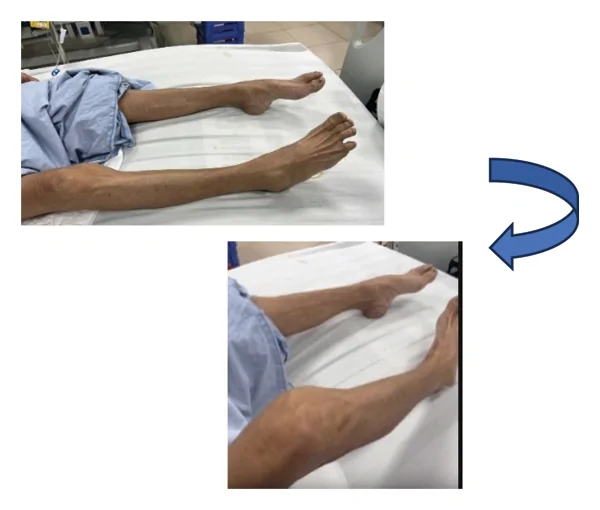
Clinical evolution of muscle rigidity, showing marked lower limb improvement after lipid emulsion therapy.

Management consisted of immediate discontinuation of the epidural infusion, along with respiratory and circulatory support, and administration of 20% ILE (Intralipid 20%). This included an initial bolus of 100 mL over 3 min (1.5 mL/kg), followed by a continuous infusion of 250 mL over 20 min (approximately 0.25 mL/kg/min; 825 mL/h) (Table 2). Laboratory investigations were performed to assess cardiac, hepatic, and renal function, along with arterial blood gas (ABG) analysis (Table 1). Within 10 min after lipid emulsion administration, the patient regained sensation in both lower limbs, muscle tone normalized, motor function returned, and she became cooperative (Figure 3).

**TABLE 2:** Recommendations for diagnosing LAST [23].

1. Classic descriptions of LAST depict a progression of subjective symptoms of CNS excitement (agitation, auditory changes, metallic taste, or abrupt onset of psychiatric symptoms), followed by seizures and then CNS depression (drowsiness, coma, or respiratory arrest). Near the end of this continuum, initial signs of cardiac toxicity (hypertension, tachycardia, or ventricular arrhythmias) are supplanted by cardiac depression (bradycardia, conduction block, asystole, decreased contractility). However, there is substantial variation in this classic description, including: + Simultaneous presentation of CNS and cardiac toxicity + Cardiac toxicity without prodromal signs and symptoms of CNS toxicity + Thus, the practitioner must be vigilant for atypical or unexpected presentation of LAST (I; B)

2. The timing of LAST presentation is variable. Immediate (< 60 s) presentation suggests intravascular injection of local anesthetic with direct access to the brain, whereas presentation that is delayed 1–5 min suggests intermittent intravascular injection, lower extremity injection, or delayed tissue absorption. Because LAST can present more than 15 min after injection, patients that receive potentially toxic doses of local anesthetic should be closely monitored for at least 30 min after injection (I; B).

3. Case reports associate LAST with underlying cardiac, neurologic, pulmonary, renal, hepatic, or metabolic disease. Heightened vigilance may be warranted in these patients, particularly if they are at the extremes of age (IIa; B).

4. The overall variability of LAST signs and symptoms, timing of onset, and association with various disease states suggests that practitioners should maintain a low threshold for considering the diagnosis of LAST in patients with atypical or unexpected presentation of CNS or cardiac signs and symptoms after receiving more than a minimal dose of local anesthetic (IIa; B).

Ventricular ectopy persisted, requiring close monitoring and intravenous amiodarone for rhythm control. Sinus rhythm was restored after 30 min, though mild tachypnea remained.

ABG analysis showed respiratory alkalosis with compensatory metabolic acidosis (pH 7.42, PCO_2_ 21 mmHg, HCO_3_^−^ 18 mmol/L, BE –10 mmol/L). Mechanical ventilation and sedation were initiated, with blood pressure maintained between 130 and 140 mmHg. Stroke and myocardial infarction were excluded; however, echocardiography revealed a marked decline in left ventricular systolic function (ejection fraction 50% vs 69% preoperatively) and mildly elevated pulmonary artery pressure (Table 3). No recurrence of LAST occurred. After 7 days, at the family’s request, she was transferred to a local healthcare facility for ongoing management.

**TABLE 3:** Perioperative imaging findings.

Date	12/01	14/01	17/01
Ejection fraction (%)	69%	50%	50%
Pulmonary artery pressure (mmHg)	Normal	40	37
Regional wall motion abnormality	None	None	None
Head CT scan	—	No lesion	—
Chest X‐ray	Normal	Normal	Normal

## 3. Method

This study is a single‐patient case report describing an episode of suspected LAST occurring in the intensive care setting following regional anesthesia with continuous local anesthetic administration. Clinical data were collected retrospectively from the patient’s medical records, including perioperative and intensive care documentation, vital signs, laboratory results, and treatment interventions. The temporal relationship between local anesthetic exposure and clinical manifestations was carefully reviewed to support the diagnosis. A focused literature review was also performed to contextualize the case. PubMed, Web of Science, ScienceDirect, and Google Scholar were searched for relevant publications on LAST, lipid emulsion therapy, and continuous local anesthetic infusion–related toxicity. Identified reports were used to support discussion and interpretation of the clinical findings.

## 4. Discussion

The incidence of LAST is relatively low, estimated at approximately 0.87–1.8 per 1000 cases, and it is exceedingly rare in the context of epidural analgesia [5, 6]. LAST primarily blocks nociceptive transmission by inhibiting sodium ion influx into axonal nerve fibers. Although their chemical structures are broadly similar, their pharmacodynamic and pharmacokinetic properties may vary depending on whether they are administered as racemic mixtures or pure enantiomers [2, 4, 7, 8].

Differential diagnoses for these clinical manifestations included drug hypersensitivity reactions, high neuraxial block, acute heart failure (including volume overload or acute myocardial infarction), and pulmonary embolism. Anaphylaxis is typically characterized by cutaneous manifestations, such as rash and bronchospasm [9]. Pulmonary embolism is classically associated with dyspnea, altered mental status, tachypnea, preserved or only mildly reduced oxygenation, tachycardia, and sometimes hypertension. Although our patient exhibited transient alterations in mental status and some cardiovascular instability, clinical assessment and investigations did not reveal predisposing factors or objective evidence supporting pulmonary embolism. We agree that concern for heart failure with possible volume overload, particularly in an elderly patient, was appropriate. This possibility was carefully considered during clinical management. However, there were no clear clinical findings supporting overt heart failure or significant volume overload. No crackles were auscultated on lung examination, and no clinical signs of acute pulmonary edema, including pink frothy sputum, were observed. In addition, fluid administration was carefully monitored and maintained in a negative fluid balance throughout the intensive care unit (ICU) stay, and no diuretic therapy was administered. High neuraxial block secondary to unrecognized epidural catheter migration into the subarachnoid space was considered in the differential diagnosis. However, careful reassessment of the epidural catheter revealed no evidence of cerebrospinal fluid (CSF) aspiration or findings suggestive of inadvertent intrathecal placement. Furthermore, the timing of symptom onset and overall clinical presentation were not consistent with a high neuraxial block, making this diagnosis less likely.

In our case, several patient and treatment‐related factors during continuous epidural analgesia likely contributed to an increased risk of LAST. *First,* advanced age is associated with altered pharmacokinetics and pharmacodynamics of LAST, including reduced clearance secondary to decreased perfusion of metabolizing organs, thereby increasing susceptibility to drug accumulation [6, 10]. In addition, age‐related neurophysiological changes, including deterioration of myelin sheaths and a decline in neuronal density, may further increase sensitivity to local anesthetic effects. Older adults frequently present with multiple comorbidities; in our patient, acute intestinal obstruction, concomitant infection, and degenerative disease may have further increased vulnerability to LAST [11]. Furthermore, skeletal muscle functions as a reservoir for local anesthetics, and age‐related sarcopenia may reduce tissue buffering capacity, thereby increasing toxicity risk [2, 6]. *Second,* prolonged continuous infusion may facilitate progressive systemic accumulation of LAST, particularly in patients with impaired metabolic reserve. Postoperative physiological changes may further alter drug disposition. Recent data have demonstrated that delayed presentation may occur at various time points, including up to several hours after the initiation of an infusion [12]. Although local anesthetics are primarily bound to alpha‐1 acid glycoprotein, altered plasma protein binding in critical illness may increase the fraction of unbound active drug; in this patient, hypoalbuminemia (26.6 g/L), together with advanced age and critical illness, may have contributed to increased susceptibility to LAST [12–14].

Severe adverse events occur in approximately 20% of patients, whereas milder manifestations may go undetected or unreported. Since the 1980s, the incidence of severe outcomes, including fatalities related to bupivacaine, has decreased, a trend attributed to improved recognition, preventive strategies, and the introduction of practice advisories in 2010 [15]. Nevertheless, LAST remains a potentially life‐threatening condition and is frequently underrecognized, particularly in intensive care settings where it may mimic other critical illnesses [7, 14]. Clinical manifestations range from mild neurological symptoms to severe cardiovascular compromise, including delirium, seizures, muscle rigidity, arrhythmias, tachycardia, hypertension, and, in severe cases, cardiovascular collapse (CC) with hypotension or cardiac arrest (Table 2). Approximately 40% of cases present with atypical features; however, seizures are the most common manifestation of central nervous system toxicity in LAST [12]. Other neurological symptoms, including perioral paresthesia, confusion, visual or auditory disturbances, dysgeusia, agitation, and decreased level of consciousness, are likely underreported [12, 14, 16].

The ratio of CC to CNS toxicity (CC/CNS ratio) represents the relative dose required to induce cardiovascular dysfunction compared with that required to provoke seizures. A lower CC/CNS ratio is associated with greater cardiotoxic potential, while a higher ratio indicates a wider safety margin. Early CNS manifestations may facilitate prompt diagnosis and treatment of LAST before CC ensues [6, 7, 17].

Levobupivacaine and ropivacaine demonstrate dose‐dependent vasoconstrictive properties that may prolong duration of action and slow systemic absorption, in contrast to bupivacaine, which has vasodilatory effects and can lead to more rapid systemic uptake [18, 19]. For this reason, newer long‐acting amide anesthetics, such as ropivacaine and levobupivacaine, were developed, both of which are considered less cardiotoxic than bupivacaine [1, 3, 20]. Bupivacaine has been reported to exhibit greater cardiotoxic and neurotoxic potential than other local anesthetics. Although the dose administered in our case was within recommended limits, a lower infusion rate than standard protocols was selected based on clinical judgment given the patient’s advanced age [4, 21, 22]. A limitation of our report is the inability to measure plasma local anesthetic concentrations. Pharmacokinetic data suggest a mean elimination half‐life of bupivacaine of approximately 5‐6 h following peripheral or neuraxial administration, with prolonged elimination reaching up to 8–10 h in some patients [21]. The risk of LAST appears to be higher with continuous infusion techniques than with single‐injection approaches, likely related to cumulative local anesthetic exposure and progressive increases in plasma concentrations [10]. Pharmacokinetic modeling by Bungart et al. [21] demonstrated clinically meaningful differences in cumulative exposure between bupivacaine and ropivacaine during prolonged continuous peripheral nerve block infusions. Their analysis suggested that bupivacaine may accumulate more rapidly, particularly under reduced clearance pharmacokinetic conditions, with divergence becoming more pronounced after 12–24 h of continuous infusion [6]. Importantly, some published dosing regimens for bupivacaine overlapped with pharmacokinetic scenarios associated with impaired clearance, suggesting a potential risk of drug accumulation in vulnerable populations, including elderly patients, those with reduced hepatic perfusion, and critically ill patients with hemodynamic instability or altered protein binding [2, 5, 12]. In these settings, systemic toxicity may not be fully avoided despite adherence to recommended dosing strategies. Considerable interindividual variability in plasma local anesthetic concentrations has also been reported, with some patients exceeding toxic thresholds. Postoperative increases in the acute‐phase reactant alpha‐1 acid glycoprotein may further alter protein binding and contribute to variability in systemic exposure and toxicity risk [1, 10]. In our patient, marked deterioration in cardiac function was observed. Given advanced age and likely pharmacokinetic alterations, including reduced clearance and altered protein binding, cumulative drug exposure may have contributed to progression toward severe LAST in this case.

**FIGURE 4:**
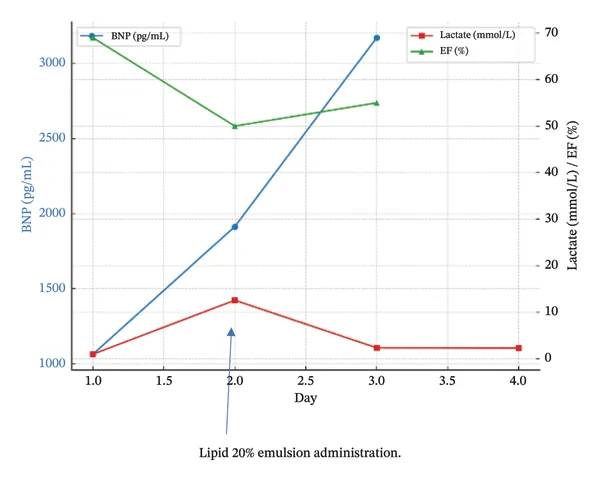
Trend of brain natriuretic peptide (BNP), lactate, and ejection fraction (EF) in a 95‐year‐old patient with suspected local anesthetic systemic toxicity.

In settings with limited resources, we did not employ invasive hemodynamic monitoring (e.g., PiCCO). Instead, we used biomarkers, such as brain natriuretic peptide (BNP), blood lactate, and echocardiography, to monitor and assess the patient’s condition, which provided a reasonable alternative (Figure 4). The cardiac function improved on echocardiography, and clinically, with stable ABG values and no trend toward increasing lactate levels, BNP continued to rise despite a negative fluid balance and the use of diuretic therapy. This may be explained by the fact that BNP release is influenced not only by intravascular volume status but also by ventricular wall stress, myocardial dysfunction, and acute hemodynamic changes. In critically ill patients, persistent myocardial injury or stress (e.g., cardiotoxicity, sepsis‐related myocardial depression, or transient dysfunction in the context of LAST) may result in delayed or dissociated BNP kinetics compared with clinical and echocardiographic improvement. Although cardiac output was preserved, adverse cardiovascular effects, including arrhythmias, conduction block, progressive hypotension, and bradycardia, remain possible. In our case, frequent ventricular premature contractions were observed. Amiodarone, the first‐line antiarrhythmic drug in this context, was administered successfully to control the arrhythmia (Table 4) [5, 10].

**TABLE 4:** Recommendations for treatment of LAST [23].

1. If signs and symptoms of LAST occur, prompt and effective airway management is crucial to preventing hypoxia and acidosis, which are known to potentiate LAST (I; B).

2. If seizures occur, they should be rapidly halted with benzodiazepines. If benzodiazepines are not readily available, small doses of propofol or thiopental are acceptable. Future data may support the early use of lipid emulsion for treating seizures (I; B).

3. Although propofol can stop seizures, large doses further depress cardiac function; propofol should be avoided when there are signs of CV compromise (III; B).

4. If seizures persist despite benzodiazepines, small doses of succinylcholine or similar neuromuscular blocker should be considered to minimize acidosis and hypoxemia (I; C).

5. If cardiac arrest occurs, we recommend standard advanced cardiac life support with the following modifications. If epinephrine is used, small initial doses (bolus 1 μg/kg boluses in the adult) are preferred (IIa; C) + Vasopressin is not recommended (III; B) + Avoid calcium channel blockers and A‐adrenergic receptor blockers (III; C) + If ventricular arrhythmias develop, amiodarone is preferred (IIa; B); treatment with local anesthetics (lidocaine or procainamide) is not recommended (III; C)

6. Lipid emulsion therapy (IIa; B): + Consider administering at the first signs of LAST, after airway management + Dosing: ‐ 1.5 mL/kg 20% lipid emulsion bolus ‐ 0.25 mL/kg per minute of infusion, continued for at least 10 min after circulatory stability is attained ‐ If circulatory stability is not attained, consider rebolus and increasing infusion to 0.5 mL/kg per minute ‐ Approximately 10 mL/kg lipid emulsion for 30 min is recommended as the upper limit for initial dosing and propofol is not a substitute for lipid emulsion (III; C).

7. Failure to respond to lipid emulsion and vasopressor therapy should prompt institution of cardiopulmonary bypass (CPB) (IIa; B). Because there can be considerable lag in beginning CPB, it is reasonable to notify the closest facility capable of providing it when CV compromise is first identified during an episode of LAST.

Recommendations from the American Society of Regional Anesthesia and Pain Medicine advise administration of an initial intravenous bolus of 20% lipid at a dose of 1.5 mL/kg over 1 minute, followed by a continuous infusion at 0.25 mL/kg/min for patients under 70 kg. The bolus dose may be repeated up to two times at 5‐min intervals, with the infusion rate increased to 0.5 mL/kg/min if adequate circulatory recovery is not achieved. The infusion should be continued until stable and adequate hemodynamic recovery is restored [7, 23]. Early recognition and timely intervention, particularly the administration of 20% lipid emulsion, can be lifesaving and reduce long‐term complications [24]. Several alternative therapeutic strategies have been explored for the management of LAST [1, 25, 26]. In our patient, neurological status improved within 10 min following lipid emulsion infusion, with recovery of lower limb motor and sensory function and a reduction in delirium. Supportive measures, such as oxygen supplementation, vasopressor therapy, or extracorporeal circulation in cases of CC, have been described; however, these approaches do not directly address the underlying mechanisms of toxicity in the same way as ILE. ILE is considered a cornerstone therapy for LAST [7, 22, 23, 25]. Although the “lipid sink” theory was initially proposed to explain its efficacy, contemporary evidence increasingly favors the “lipid shuttle” mechanism [11]. In this model, lipophilic local anesthetics are redistributed from highly perfused target organs, particularly the heart and brain, to peripheral compartments, such as the liver, skeletal muscle, and adipose tissue, thereby reducing tissue toxicity and facilitating metabolism and elimination. Beyond drug redistribution, ILE may exert direct cardiometabolic effects, including improved myocardial energy substrate availability, enhancement of cardiac contractility, and partial reversal of sodium channel blockade. These combined mechanisms may contribute to the rapid hemodynamic recovery observed following ILE administration in patients with LAST [5, 11, 27]. In summary, our case shares similarities with previous reports of LAST successfully treated with ILE, but it also highlights the importance of comprehensive postoperative monitoring and vigilance for potential complications of local anesthetics. This is particularly relevant in critically ill patients in the ICU, where LAST may be misdiagnosed as other severe conditions. Even when symptoms improve with ILE, the possibility of toxicity should not be overlooked. Future studies should continue to refine dosing regimens and adjunctive management strategies targeting cardiovascular function, while reinforcing ILE as the cornerstone of LAST treatment.

## 5. Conclusion

Epidural analgesia is widely applied in anesthesiology and critical care, and has been proven highly effective, particularly in abdominal surgery [17]. However, LAST during epidural analgesia is a complication that warrants vigilance, especially in elderly patients with impaired clearance and drug accumulation, even when recommended dosing guidelines are strictly followed, as age‐related physiological changes may increase susceptibility to LAST. Early recognition and treatment with ILE can lead to favorable recovery. Close monitoring of patients receiving local anesthetics remains essential for the timely prevention and management of serious complications.

**Nomenclature**
ILE: Intravenous lipid emulsion
ERAS: Enhanced recovery after surgery
LAST: Local anesthetic systemic toxicity
AST: Aspartate aminotransferase
ALT: Alanine aminotransferase
LA: Local anesthetics
ICU: Intensive care unit
CFS: Cerebrospinal fluid
CC: Cardiovascular collapse
CNS: Central nervous system
ABG: Arterial blood gas
BNP: Brain natriuretic peptide

## Author Contributions

Thi Anh Tuyet Cao was involved in the perioperative anesthetic management of this patient. Thi Anh Tuyet Cao and Thang Toan Nguyen collected the clinical data and drafted the manuscript as major contributors and critically reviewed and revised the manuscript.

## Funding

This research received no external funding.

## Ethics Statement

The study was conducted in accordance with the Declaration of Helsinki (1975, revised in 2013) and was approved by the Ethics Committee of the University of Magdeburg (approval code: R06‐25).

## Consent

Written informed consent was obtained from the patient for publication of this case report and any accompanying images. A copy of the written consent is available for review by the Editor‐in‐Chief of this journal.

## Conflicts of Interest

The authors declare no conflicts of interest.

## Supporting Information

Additional supporting information can be found online in the Supporting Information section.

## Supporting information


**Supporting Information** Check CARE.

## Data Availability

The original contributions presented in this study are included in the article. Further inquiries can be directed to the corresponding author.
